# Endoscopic Treatment and Considerations for a Rare of Killian‐Jamieson Diverticulum: A Case Report

**DOI:** 10.1002/jgh3.70127

**Published:** 2025-03-05

**Authors:** Guoyao Sun, Wen Jia, Zhuo Yang, Jiao Liu, Yong Sun

**Affiliations:** ^1^ Department of Endoscopy General Hospital of Northern Theater Command Shenyang Liaoning China

**Keywords:** endoscopic ultrasound, Killian‐Jamieson diverticulum, Laimer's diverticulum, pharyngoesophageal diverticula, Zenker's diverticulum

## Abstract

**Background:**

Pharyngoesophageal diverticulum, including the most common Zenker's diverticulum, Killian‐Jamieson diverticulum, and the rarer Laimer's diverticulum, require accurate differentiation for proper treatment. This case report explores the endoscopic features and the diagnostic and therapeutic value of endoscopic ultrasound (EUS) in the management of Killian‐Jamieson diverticulum.

**Case Presentation:**

A 57‐year‐old woman presented with dysphagia for solids over the past three months. Esophagogastroduodenoscopy (EGD) revealed an esophageal diverticulum located below the upper esophageal sphincter. EUS measured the diverticulum to be 1.0 cm by 1.4 cm and confirmed the absence of muscularis propria, thereby ruling out Laimer's diverticulum. The patient underwent flexible endoscopic septum division (FESD) without any adverse events. A barium esophagram three months post‐surgery indicated a left‐sided Killian‐Jamieson diverticulum.

**Conclusion:**

This case highlights the importance of combining endoscopic features with barium swallow and EUS for accurate diagnosis of Killian‐Jamieson diverticulum. The results suggest that such an integrated diagnostic approach enhances diagnostic accuracy and guides treatment, offering insights into the management of pharyngoesophageal diverticulum.

## 1 Introduction

**FIGURE 1:**
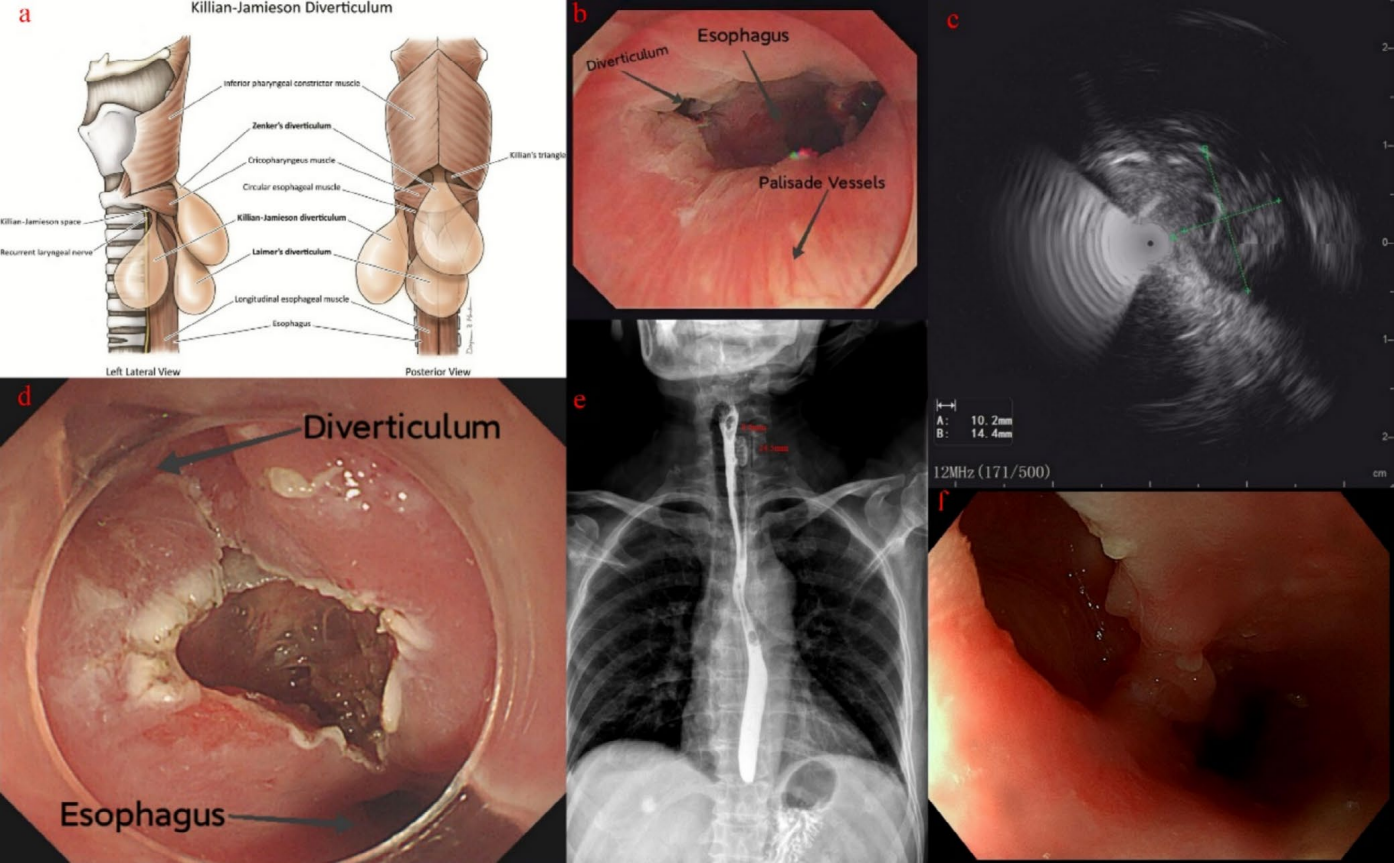
Anatomical structure and endoscopic and imaging features of Killian‐Jamieson diverticulum. (a) The anatomical structure of the pharyngoesophageal diverticulum. (b) The diverticulum is located beneath the palisade vessels. (c) EUS indicates that the diverticulum measures 1.0 cm in length and 1.4 cm in width and lacks the muscularis propria. (d) An incision was made at the midpoint of the septum. (e) Three months after direct septotomy, barium contrast imaging revealed a diverticulum on the left side, measuring 2.45 cm in height and 0.8 cm in length. (f) One year after direct septotomy, EGD revealed a wide‐mouthed diverticulum.

Pharyngoesophageal diverticulum can be classified into the most common Zenker's diverticulum, the Killian‐Jamieson diverticulum, and the rarest form, Laimer's diverticulum (Figure 1a [1]). Currently, there is no endoscopic description of Laimer's diverticulum [2]. The first two types are pseudodiverticula, consisting only of the mucosal and submucosal layers. In contrast, Laimer's diverticulum is a true diverticulum, containing all layers of the esophageal wall. We report a case of a 57‐year‐old female with Killian‐Jamieson diverticulum, summarizing its endoscopic features and the potential diagnostic value of EUS. This will assist endoscopists in distinguishing between the three types of diverticula.

## 2 Case Report

A 57‐year‐old woman had experienced symptoms of dysphagia for solid foods over the past 3 months. Under esophagogastroduodenoscopy (EGD) visualization, we identified an esophageal diverticulum. Its distance from the dental arches is approximately 20–22 cm, located below the typical Zenker's diverticulum and situated beneath the palisade vessels corresponding to the upper esophageal sphincter (Figure 1b), which rules out the possibility of a Zenker's diverticulum. The diverticulum was located between the 9 o'clock and 10 o'clock positions. After switching to the EUS, the diverticulum was measured to be approximately 1.0 cm in length and 1.4 cm in width (Figure 1c). The EUS also revealed the absence of muscularis propria, which excluded Laimer's diverticulum. Based on the endoscopic characteristics of the diverticulum, we initially identified it as a Killian‐Jamieson diverticulum. The patient underwent flexible endoscopic septum division (FESD). Food debris within the diverticulum was cleared using forceps. An incision was made at the midpoint of the septum, and a complete septotomy was performed along its longitudinal axis (Figure 1d) by Gold Knife (Micro‐Tech Co. Ltd., Nanjing, China). After confirming the absence of esophageal mucosal perforation, the incision was closed using five clips. Three months later, the patient reported significant symptom improvement and underwent a barium esophagram. The results indicated a left‐sided Killian‐Jamieson diverticulum (Figure 1e). One year later, the patient underwent a follow‐up EGD, revealing a wide‐mouthed diverticulum (Figure 1f).

## 3 Discussion

Yuki Watanabe et al. reported a case of Killian‐Jamieson Diverticulum diagnosed as Zenker's Diverticulum prior to surgery [3]. Accurate diagnosis of diverticulum types can only be ensured by fully exposing the tissue structures during open surgery. This suggests that relying solely on barium swallow examinations may have limitations in certain endoscopic treatments. The upper palisade vessels can be used to estimate the level of the upper esophageal sphincter, aiding in the differentiation between Zenker's diverticulum located above the upper esophageal sphincter and the Killian‐Jamieson diverticulum and Laimer's diverticulum located below the upper esophageal sphincter [4]. Under EGD visualization, the Killian‐Jamieson diverticulum can be observed between the 9 o'clock and 10 o'clock positions, whereas Zenker's diverticulum is typically located at the 6 o'clock position [5]. According to anatomical structure, Laimer's diverticulum should also be located at the 6 o'clock position. Killian‐Jamieson diverticulum is significantly smaller on average, with an average size of 1.9 cm compared to 2.5 cm for Zenker's diverticulum [6]. EUS not only aids in assessing the size of the diverticulum but also helps detect the absence of the muscularis propria, thereby distinguishing between true diverticulum and pseudodiverticula. Based on the above endoscopic features and combined with EUS, the type of pharyngoesophageal diverticulum can be preliminarily identified. Additionally, barium swallow examinations can further improve diagnostic accuracy. The endoscopic features of Laimer's diverticulum summarized in this article may assist endoscopists in detecting the first case of Laimer's diverticulum and provide initial guidance for its diagnosis in the future. In addition to the FESD technique used in this case, the recent introduction of KJ‐POEM, an adaptation of the original Z‐POEM technique, has further advanced the endoscopic treatment of Killian‐Jamieson diverticulum [7, 8]. Moving forward, our center will actively explore KJ‐POEM‐related techniques.

In conclusion, for pharyngoesophageal diverticula treated endoscopically, combining barium swallow and endoscopic features can enhance diagnostic accuracy. This case emphasizes the importance of a comprehensive diagnostic approach.

## Ethics Statement

All procedures conducted were in compliance with the ethical principles outlined in the Declaration of Helsinki and its subsequent revisions.

## Consent

Written informed consent was obtained from the patient for the publication of this case report and accompanying images.

## Conflicts of Interest

The authors declare no conflicts of interest.
